# Supplementary material for: Wild Wheat Rhizosphere-Associated Plant Growth-Promoting Bacteria Exudates: Effect on Root Development in Modern Wheat and Composition
Source: Int J Mol Sci. 2022 Dec 3;23(23):15248. doi: 10.3390/ijms232315248 (PMC9740669; doi:10.3390/ijms232315248)
Supplement: Supplementary file 1 [file ijms-23-15248-s001.zip › Method S1_IJMS.pdf]

SUPPORTING INFORMATION FOR EXPERIMENTAL PROCEDURES

Supplementary LCMS based metabolomics: equipped with a heated electrospray probe (HESI II) coupled to a U-HPLC Ultimate 3000 RSLC system (Thermo Fisher Scientific, Hemel Hempstead, U.K.). Separation was done on a Luna Omega Polar C18 column (150 mm × 2.1 mm i.d., 1.6 μm, Phenomenex, Sartrouville, France) equipped with a guard column. Mobile phase A (MPA) was water with 0.05% formic acid (FA), and mobile phase B (MPB) was acetonitrile with 0.05% FA. The solvent gradient was 0 min, 100% MPA; 1 min, 100% MPA; 22 min, 100% MPB; 25 min, 100% MPB; 25.5 min, 100% MPA; 28 min, 100% MPA. The flow rate was 0.3 mL/min, the column temperature was set to 40 °C, autosampler temperature was set to 5 °C, and injection volume fixed to 5 μL. Mass detection was performed in positive (PI) ionization mode at 35 000 resolving power [full width at half-maximum (fwhm) at 400 m/z] for MS1 and 17 500 for MS2 with an automatic gain control (AGC) target of 1×10^6 for full scan MS1 and 1×10^5 for MS2. Ionization spray voltages were set to 3.5 kV, and the capillary temperature was set to 256 °C. The mass scanning range was m/z 100−1500. Each full MS scan was followed by data-dependent acquisition of MS/MS spectra for the six most intense ions using stepped normalized collision energy of 20, 40, and 60 eV.

UHPLC-HRMS raw data were processed with MS-DIAL version 4.70 (Tsugawa *et al.* 2015) for mass signal extraction between 100 and 1500 Da from 0.5 to 18.5 min. Respectively MS1 and MS2 tolerance were set to 0.01 and 0.05 Da in centroid mode. The optimized detection threshold was set to 1.5×10^6 concerning MS1 and 10 for MS2. Peaks were aligned on a quality control (QC: pool of all samples) reference file with a retention time tolerance of 0.1 min and a mass tolerance of 0.015 Da. Peak annotation was performed with an in-house database built on MS-FINDER model (Tsugawa et al. 2016).

MS-DIAL data were then cleaned with the MS-CleanR workflow (Fraisier-Vannier *et al.* 2020) by selecting all filters with a minimum blank ratio set to 0.8, a maximum relative standard deviation (RSD) set to 40, and a relative mass defect (RMD) ranging from 50 to 3 000. The maximum mass difference for feature relationships detection was set to 0.005 Da, and the maximum RT difference to 0.025 min. The Pearson correlation links were considered with correlation ≥0.8 and statistically significant $\alpha = 0.05$. Two peaks were kept in each cluster to with the most intense and the most connected. The kept features were annotated with MS-FINDER version 3.52. The MS1 and MS2 tolerances were respectively set to 5 and 15 ppm. Formula finder were exclusively processed with C, H, O, N, P, and S atoms. Databases (DBs) based on *Pseudomonas* and *Anterobacter* (genus), were constituted with the dictionary of natural product (DNP, CRC press, DNP on DVD v. 28.2). The internal generic DBs from MS-FINDER used were KNApSAcK, PlantCyc, HMDB, LipidMaps, NANPDB,NPatlas and UNPD. Annotation prioritization was done by ranking *genus* DB, followed by generic DBs using the final MS-CleanR step.
