# Supplementary material for: Wild Wheat Rhizosphere-Associated Plant Growth-Promoting Bacteria Exudates: Effect on Root Development in Modern Wheat and Composition
Source: Int J Mol Sci. 2022 Dec 3;23(23):15248. doi: 10.3390/ijms232315248 (PMC9740669; doi:10.3390/ijms232315248)
Supplement: Supplementary file 1 [file ijms-23-15248-s001.zip › Supplemental figures.ijms.pdf]

Supplemental figures

(A) *BPMP-PU-28*

Chromosome

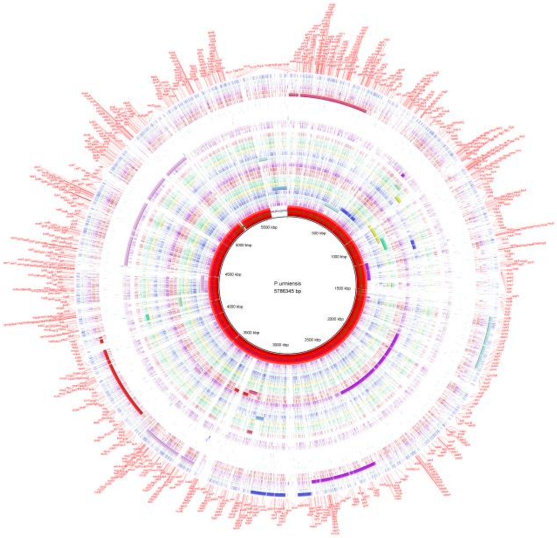

(B) *BPMP-EL-40*

Chromosome

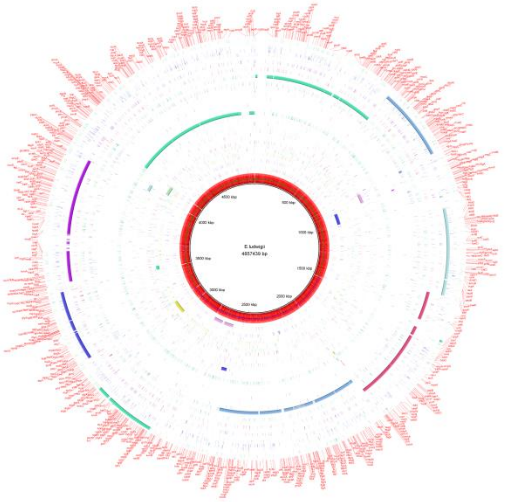

Plasmid

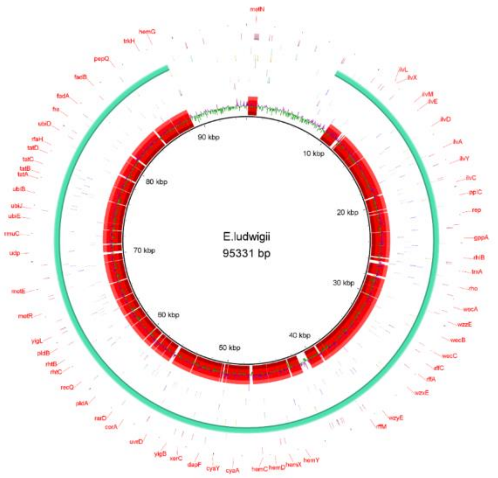

Figure S1. Genome maps of *BPMP-PU-28* and *BPMP-EL-40* bacterial strains.

Blast Ring Image Generator (BRIG) diagram showing homologous chromosome and plasmid segments resulting from the sequencing of the *BPMP-PU-28* (**A**) and *BPMP-EL-40* (**B**) genomes. The genomic sequences were blasted against the reference genome of *Pseudomonas urmiensis* (JABWRE020000001.1) and *Enterobacter ludwigii* (CP017279.1, CP017280.1) of the NCBI.

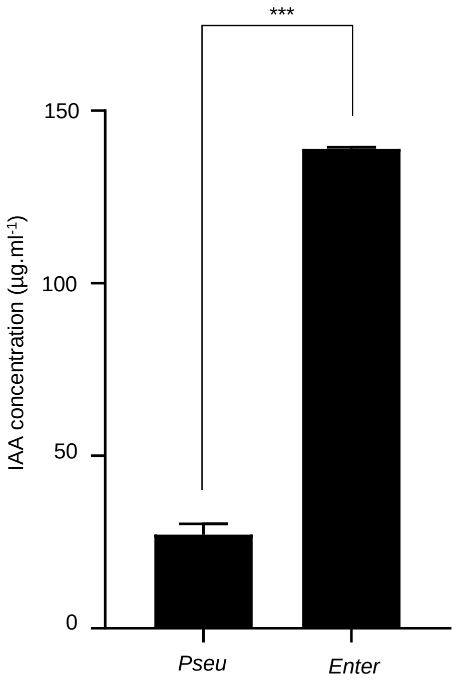

Figure S2. Indole-3-acetic acid (IAA) production by *BPMP-PU-28 (Pseudomonas urmiensis)* and *BPMP-EL-40 (Enterobacter ludwigii)*.

The bacteria were grown for 48 h in LB medium supplemented with tryptophan (0.2%). IAA was assayed in the media as described in [105,106]. Means ± SE (n = 3). *** above the bars indicate that the difference between *BPMP-PU-28 (Pseu: Pseudomonas urmiensis) and BPMP-EL-40 (Enter: Enterobacter ludwigii)* is statistically significant (Student's t-Test, P≤ 0.001).

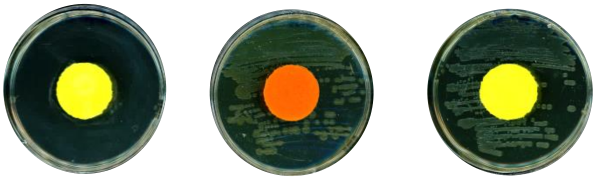

Control ***Pseu*** ***Enter***

Figure S3. Hydrogen cyanide production ability of *BPMP-PU-28 (Pseudomonas urmiensis)* and *BPMP-EL-40 (Enterobacter ludwigii)*. Both *BPMP-PU-28* (*Pseu: Pseudomonas urmiensis*) and *BPMP-EL-40* (*Enter: Enterobacter ludwigii*) were grown on agar plates (LB medium supplemented with 0.25% glycine). A change in color from yellow to orange of a piece of filter paper soaked in alkaline picrate solution and adhering to the inside of the Petri dish lid indicates hydrogen cyanide production capacity. Control: agar plate without bacteria.

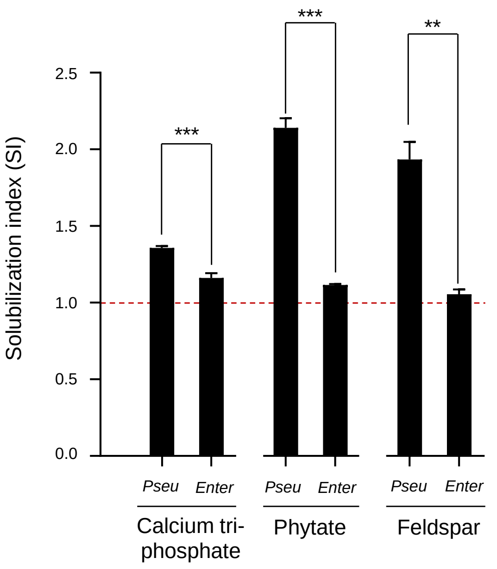

Figure S4. Ability of *BPMP-PU-28 (Pseudomonas urmiensis)* and *BPMP-EL-40 (Enterobacter ludwigii)* to use poorly available sources of phosphorus and potassium.

The two bacterial strains were grown on agar plates containing a poorly available source of P, either tri-calcium phosphate or phytate, in Pikovskaya medium, or a poorly soluble source of K^+, feldspar (potassium aluminosilicate, Potash Feldspar, Bath Potters' Supplies, Somerset, UK) in Alexandrov medium. Bacterial ability to use these nutrient sources was assessed by determining the so-called solubilisation index (SI), based on measurement of the diameter of the bacterial colony and of the diameter of the halo zone around the colony (at 5 days of growth after the drop of the bacterial suspension on the agar plate). A SI value higher than 1 indicates the ability of bacteria to solubilize the nutrient source. Means ± SE (n = 3). ** and *** above the bars indicate that the difference between the bacterial strains *BPMP-PU-28 (Pseu: Pseudomonas urmiensis)* and *BPMP-EL-40* (*Enter: Enterobacter ludwigii*) is statistically significant (Student's t-Test, P≤ 0.01 and 0.001 respectively).

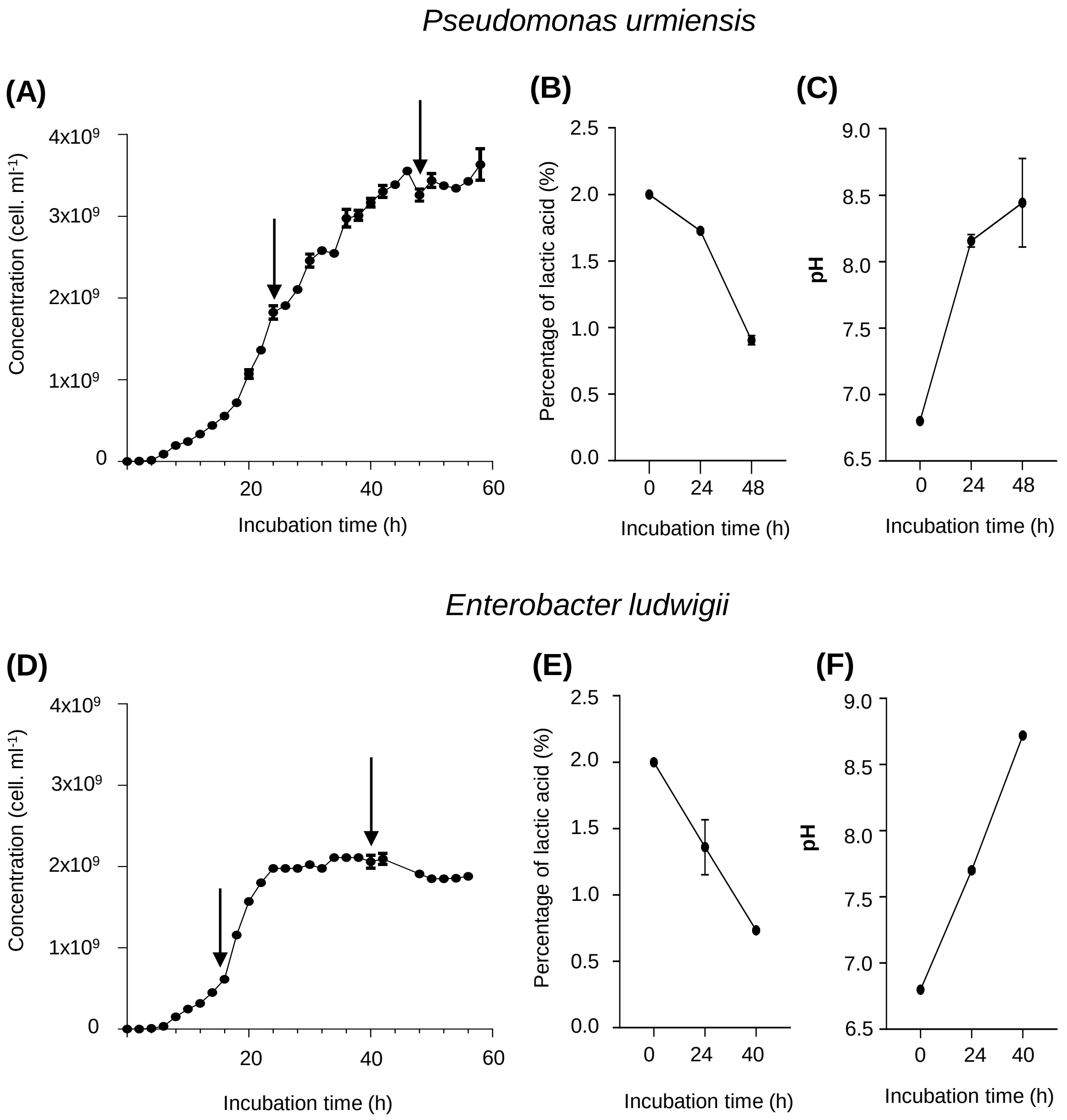

Figure S5. Growth of *BPMP-PU-28 (Pseudomonas urmiensis)* and *BPMP-EL-40 (Enterobacter ludwigii)* in a minimal medium supplemented with lactate as carbon source.

Composition of the growth medium: Hoagland solution supplemented with 2% (ca. 220 mM) lactate, pH 6.8. Bacteria were grown for 60 hours at 37°C and 200 rpm.

(**A**) and (**D**) Typical growth kinetics of *Pseudomonas urmiensis and Enterobacter ludwigii*. The arrows above the kinetics indicate the times at which aliquots of culture media were taken in similar experiments (during the exponential and stationary phases, E and S, respectively) for lactate concentration, pH measurement, and for metabolomics and proteomics analyses of bacterial culture supernatants (see Figures 5 and 6) and tests of their effects on root system and root hair development (Figures 3 and 4).

(**B**) and (**E**) Decrease in the percentage of lactate of the culture medium of *Pseudomonas urmiensis* (**B**) and *Enterobacter ludwigii* (**E**).

(**C**) and (**F**) Evolution of the pH in the *Pseudomonas urmiensis* (**C**) and *Enterobacter ludwigii* (**F**) culture medium.

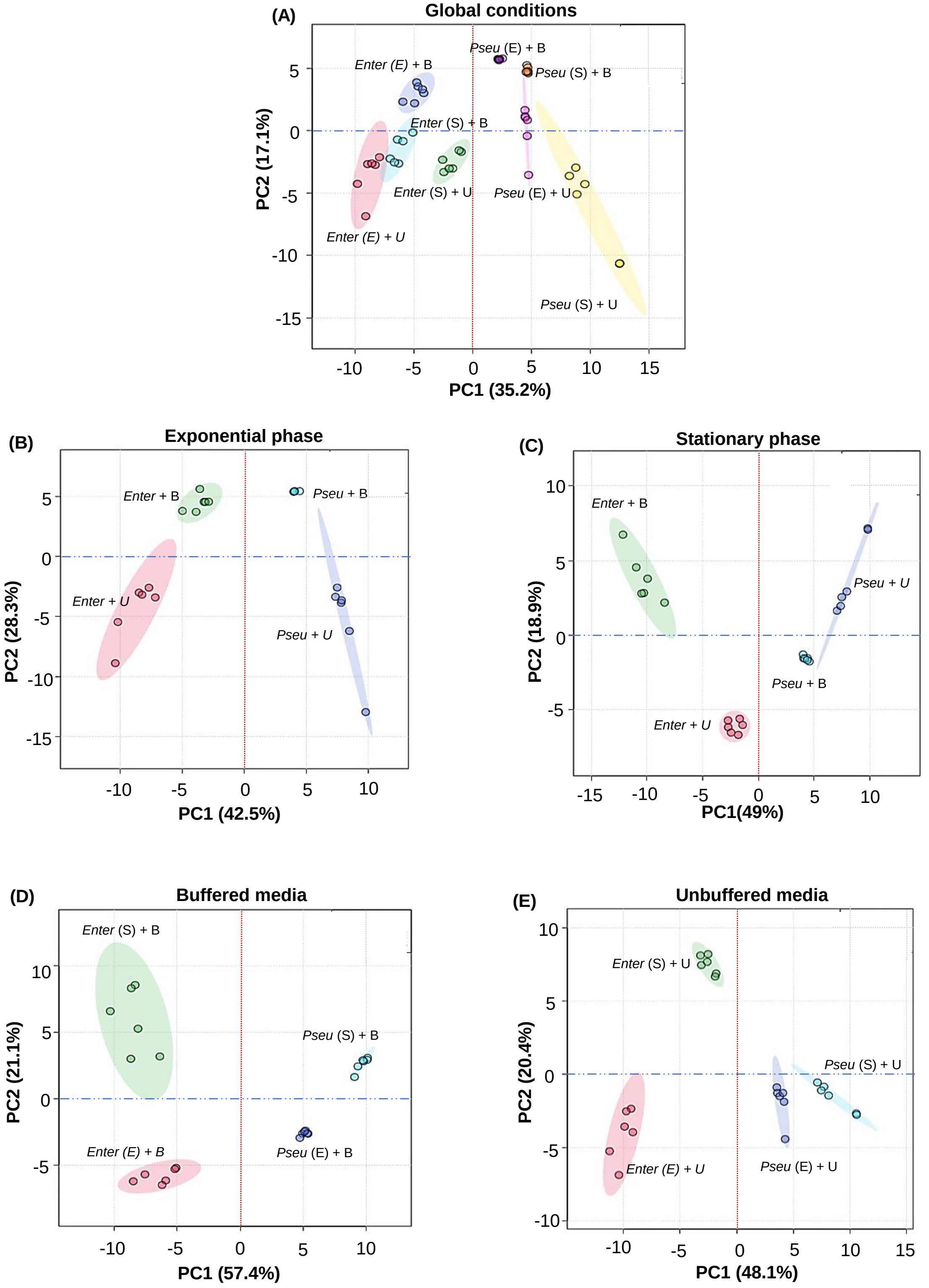
(A)
Global conditions
Enter (E) + B
Pseu (E) + B
Pseu (S) + B
Enter (S) + B
Enter (S) + U
Pseu (E) + U
Enter (E) + U
Pseu (S) + U
PC2 (17.1%)
PC1 (35.2%)
(B)
Exponential phase
Enter + B
Pseu + B
Enter + U
Pseu + U
PC2 (28.3%)
PC1 (42.5%)
(C)
Stationary phase
Enter + B
Pseu + U
Pseu + B
Enter + U
PC2 (18.9%)
PC1(49%)
(D)
Buffered media
Enter (S) + B
Pseu (S) + B
Enter (E) + B
Pseu (E) + B
PC2 (21.1%)
PC1 (57.4%)
(E)
Unbuffered media
Enter (S) + U
Pseu (S) + U
Enter (E) + U
Pseu (E) + U
PC2 (20.4%)
PC1 (48.1%)

Figure S6. Comparative metabolomics profiling of supernatants of *BPMP-PU-28 (Pseudomonas urmiensis)* and *BPMP-EL-40 (Enterobacter ludwigii)* culture media: principal component analysis (PCA) of the metabolites identified in the culture supernatant of the two bacterial strains.

BPMP-PU-28 and BPMP-EL-40 bacteria (*Pseu: Pseudomonas urmiensis* and *Enter: Enterobacter ludwigii*, respectively) were grown (6 biological replicates per condition) in a minimal Hoagland medium complemented with 2% lactate (as carbon source) and buffered with phosphate buffer (150 mM, pH 6.8) or unbuffered (B and U, respectively). Aliquots of culture media were collected during the exponential (E) or stationary phases (S) (as indicated by the arrows in Figure S5 A and D*)*. Untargeted metabolomics analyses of the collected culture supernatant were performed via UHPLC-MS/MS fragmentation. Score plots were generated using MetaboAnalyst software. (**A**) Global PCA compiling both strains at two different stages (E and S) and in the two culture media (B and U). (**B**) and (**C**) PCA analysis of the growth media collected during the exponential (**B**) and stationary phase (**C**) of the two strains in presence of buffered or unbuffered media. (**D**) and (**E**) PCA analysis of the buffered (**D**) and unbuffered (**E**) culture media collected for each of the two strains during the exponential and stationary phases (E and S).

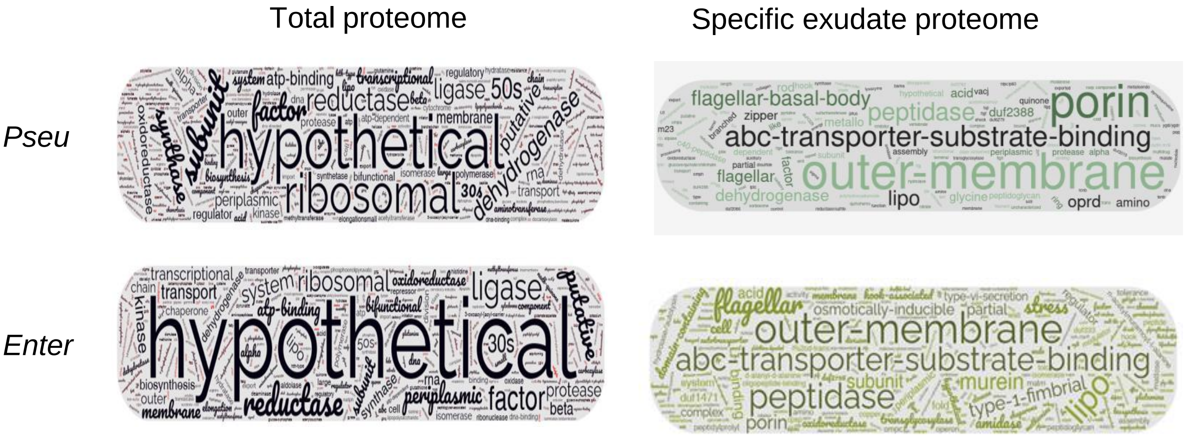

Figure S7. Tag cloud comparison between the total proteome and exudate-specific proteome in *BPMP-PU-28 (Pseudomonas urmiensis)* and *BPMP-EL-40 (Enterobacter ludwigii)*.

BPMP-PU-28 (Pseu: Pseudomonas urmiensis) and *BPMP-EL-40 (Enter: Enterobacter ludwigii)* were grown in minimal buffered medium. Culture samples (bacteria and culture supernatants isolated by centrifugation) were collected during the stationary phase (see Figure S5) for proteomics analyses (total bacterial proteomes and exudate-specific proteomes, respectively). Lists of proteins: see Table S5. The annotations of the proteins found in the total proteomes and in the exudate-specific proteomes of the two bacterial strains were used to generate tag clouds (https://www.nuagesdemots.fr/)

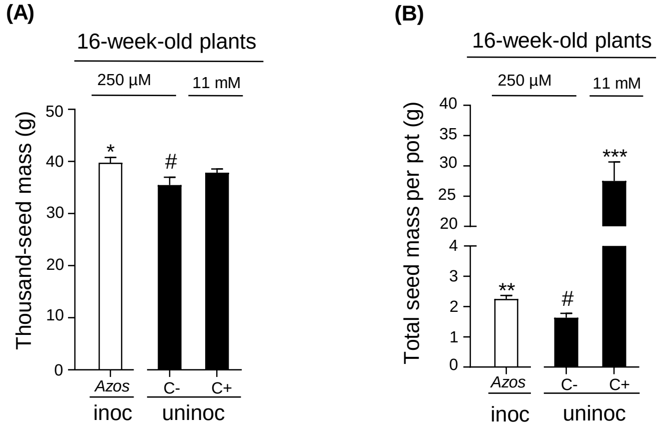

Figure S8. Effect of inoculation with *Azospirillum sp. (Azos)* on seed yield of wheat plants grown under low assimilable nitrogen availability.

Elite durum wheat (cv. Anvergur) plants were grown in greenhouse in pots on an artificial solid substrate (6 pots per condition, 4 plants per pot). They were inoculated with *Azospirillum sp. (Azos)* or not inoculated. Nutrients necessary for plant growth were brought with 2 different watering solutions, all derived from Hoagland medium but containing either 250 µM assimilable nitrogen (provided as NO_3^-) for inoculated plants (white bars) and for non-inoculated "negative" control plants (C-, black bars), or containing 11 mM assimilable nitrogen (10 mM NO_3^- and 1 mM NH_4^+) for non-inoculated "positive" control plants (C+, black bars). Thousand-seed mass (**A**) and total seed mass per pot (**B**) of 16-week-old plants. Means ± SE (n = 6 pots). *, ** and *** above the bars indicate that the difference with the uninoculated condition under nitrogen limitation (#) is statistically significant (Student's t-Test, P≤ 0.05, 0.01 and 0.001 respectively).
