# Supplementary material for: Wild Wheat Rhizosphere-Associated Plant Growth-Promoting Bacteria Exudates: Effect on Root Development in Modern Wheat and Composition
Source: Int J Mol Sci. 2022 Dec 3;23(23):15248. doi: 10.3390/ijms232315248 (PMC9740669; doi:10.3390/ijms232315248)
Supplement: Supplementary file 1 [file ijms-23-15248-s001.zip › Supplemental.tables.ijms_.pdf]

Table S1. Physicochemical properties of the soil from the Lebanon refuge area where the endemic wild emmer was selected, and from the French soil where we grew this wheat ancestor.

We isolated diazotrophic bacteria from the rhizospheric soil of this wild emmer growing in the Lebanon refuge area and in the French soil.

Physical properties

	Lebanese soil	French soil
**Sand (%)**	41	33
**Silt (%)**	39	40
**Clay (%)**	20	27
**Soil textural class**	Alluvial clay	Alluvial clay

	Lebanese soil		French soil	
**Parameters**	**Values**	**Remarque**	**Values**	**Remarque**
**pH (H_2O)**	7	Neutral	7.5	Basic
**EC ($ds.m^{-1}$)**	0.13	Low	0.2	Low
**Organic matter (%)**	2.1	Medium	1.76	Medium
**Total limestone (%)**	8	Medium	3	Medium
**Active limestone (%)**	2.7	Low	1	Low
**Organic nitrogen (%)**	0.13	Low	0.11	Low
**Available P (ppm)**	51.2	Medium	104	Medium
**Available K (ppm)**	544	High	413	High
**Exchangeable Na (ppm)**	118	Low	165	Low
**Exchangeable Mg (ppm)**	665	High	468	High
**Exchangeable Ca (ppm)**	10126	High	7502	High

Table S2. (Data sheet)

List of metabolites identified in *BPMP-PU-28* (*Pseudomonas urmiensis*) and *BPMP-EL-4*0 (*Enterobacter ludwigii*) culture supernatants.

Both bacteria were grown in buffered minimal medium and aliquots of bacterial culture supernatant were collected during the stationary phase.

Table S3. Global comparative metabolomics profiling of *BPMP-PU-28 (Pseudomonas urmiensis)* and *BPMP-EL-4*0 (*Enterobacter ludwigii*) culture supernatants: top 50 more discriminating metabolites in buffered or unbuffered culture media collected during the exponential or stationary phases.

For the complete list of metabolites, see Table S2. Corresponding heat map: see Figure 6A. As described in the legends to Figure S5 and Figure 6A, the bacteria were grown either in buffered (B) or unbuffered (U) minimal medium and aliquots of bacterial culture supernatant were collected either during the exponential (E) or stationary (S) phase (see Figure S5) for metabolomics analysis. Numbers on the left refer to the metabolites whose distribution and abundance are reported in the heat map shown in Figure 5a. Annotations were performed using MSCleanR workflow. N°: metabolite number; Rt min: retention time in min; m/z[M+H]+: mass/charge; bank: bank used to identify the metabolite; total score: score of the fragmented metabolite (only metabolites identified with scores equal to or higher than 6.5 were selected). Three biological replicates, each one being used for two technical replicates.

N°	Rt.min	m/z [M+H] +	Bank	Formula	Compound annotated	Total score
10	6.124	275.10382	Pseudo	$C_{14}H_{16}N_2O_4$	Tryptophan; N?-Methoxycarbonyl, Me ester	6.935
27	6.232	216.12376	Generic	$C_{10}H_{19}NO_4$	(2S)-5-acetamidopentyl 2-hydroxypropanoate	7.2705
201	9.614	185.11804	Generic	$C_{10}H_{18}O_3$	8-methyl-5-oxo-nonanoic acid	7.2146
502	5.374	227.1393	Generic	$C_{11}H_{18}N_2O_3$	Cyclo-(L-Pro-4-OH-L-Leu)	7.0225
29	9.028	187.13348	Pseudo	$C_{10}H_{20}O_3$	?-hydroxydecanoic acid	6.842
7	7.053	159.10223	Pseudo	$C_8H_{16}O_3$	(+/-)-3-hydroxyoctanoic acid	8.3247
17	5.511	215.1394	Generic	$C_{10}H_{18}N_2O_3$	Dethiobiotin	7.3222
393	9.14	160.07588	Pseudo	$C_{10}H_9NO$	2-methyl-1H-quinolin-4-one	6.513
26	8.504	183.10196	Pseudo	$C_{10}H_{14}O_3$	[2-(5-Oxo-1-cyclopenten-1-yl)methyl]butanoic acid	7.2577
6	11.626	351.21487	Generic	$C_{18}H_{32}O_5$	Corchorifatty acid F	6.6124
451	8.338	506.25381	Generic	$C_{27}H_{39}NO_6S$	9-Oxoepothilone D	6.794
51	11.688	310.14432	Generic	$C_{19}H_{19}NO_3$	CJ 16171	7.077
108	9.168	468.27307	Generic	$C_{28}H_{37}NO_5$	Pyxipyrrolone B	6.9859
32	4.006	405.10495	Pseudo	$C_{17}H_{18}N_4O_8$	1,2,3,4,7,8-Hexahydro-6-(4-hydroxyphenyl)-8-(2,3,4,5-tetrahydroxypentyl)-2,4,7-pteridinetrione	6.5099
296	10.344	312.16003	Generic	$C_{19}H_{21}NO_3$	CJ 16170	7.5184
380	8.095	357.11646	Generic	$C_{16}H_{20}O_9$	Pestalotiopyrone K	7.3357
66	6.534	435.11423	Generic	$C_{24}H_{20}O_8$	Terfestatin C	6.8471
1	7.555	275.11386	Generic	$C_{12}H_{20}O_7$	Methyl (3r)-3-{[(3r)-3-{[(3r)-3-hydroxybutanoyl]oxy}butanoyl]oxy}butanoate	7.058
450	9.711	501.1954	Generic	$C_{23}H_{32}O_{12}$	Talapolyester D	7.2782
445	9.075	487.17969	Generic	$C_{22}H_{30}O_{12}$	Talapolyester A	7.0784
403	7.653	387.1636	Generic	$C_{18}H_{26}O_9$	Macrosphelide K	7.1868
514	7.244	245.13892	Generic	$C_{12}H_{20}O_5$	Phomolide G	7.2239
498	6.141	217.10753	Generic	$C_{10}H_{16}O_5$	Aspyronol	7.0509
427	6.836	437.14267	Generic	$C_{21}H_{24}O_{10}$	Daldinin D	6.9164
223	7.286	217.10777	Pseudo	$C_{10}H_{18}O_5$	3-hydroxysebacate	7.5995
378	6.782	357.1163	Generic	$C_{16}H_{20}O_9$	Pestalotiopyrone K	7.4888
346	8.306	313.12631	Generic	$C_{15}H_{20}O_7$	Cyclocalopin A-15-OL	7.7123
288	5.4	305.08792	Pseudo	$C_{12}H_{18}O_9$	Galactose; 1,2,6-Tri-Ac	6.6442
382	5.596	359.10474	Generic	$C_{11}H_{23}N_2O_7PS$	Pantetheine 4'-phosphate	6.7873
447	9.718	487.14368	Generic	$C_{28}H_{22}O_8$	Streptoanthraquinone A	6.7253
93	8.262	473.12738	Generic	$C_{19}H_{22}N_4O_9$	Nikkomycin Wx	6.862
500	3.716	259.07953	Pseudo	$C_9H_{16}O_7$	Caryose	6.6703
366	7.956	343.10049	Pseudo	$C_{13}H_{20}O_9$	Methyl ?-D-glucopyranoside; 2,4,6-Tri-Ac	7.609
43	11.924	243.19626	Pseudo	$C_{14}H_{28}O_3$	(+/-)-3-hydroxymyristic acid	6.9071
341	9.204	310.23834	Generic	$C_{18}H_{31}NO_3$	N-Tetradecenoyl-L-homoserine lactone	6.5151
348	7.213	313.12659	Generic	$C_{15}H_{20}O_7$	Cyclocalopin A-15-OL	6.746
217	10.189	211.16957	Pseudo	$C_{13}H_{22}O_2$	2,4-Tridecadienoic acid	6.5514
414	10.748	415.21228	Generic	$C_{24}H_{30}O_6$	Chaetopenoid A	7.1278
67	6.062	245.09293	Pseudo	$C_{13}H_{14}N_2O_3$	N-acetyl-D-tryptophan	6.7931
519	5.952	273.16797	Pseudo	$C_{14}H_{24}O_5$	Mupirocin H	6.8823
351	7.624	315.17844	Generic	$C_{16}H_{26}O_6$	Isoaigialone A	6.5883
363	6.861	331.2099	Generic	$C_{17}H_{30}O_6$	Linieodolide A	7.3222
497	9.597	214.25349	Generic	$C_{14}H_{31}N$	Medelamine A;NK 148198A	7.3576
505	5.302	118.08648	Generic	$C_{11}H_{19}NO_8$	N-Acetylmuramate	6.5535
478	4.047	111.08068	Generic	$C_7H_{10}O$	2-Isopropylfuran	6.751
528	5.263	269.06424	Generic	$C_{11}H_{10}N_4O_3$	Not named	7.3162
413	8.949	415.1221	Pseudo	$C_{18}H_{22}O_{11}$	Dihydro-4-hydroxy-2(3H)-furanone; O-[4-Hydroxy-3-methoxybenzoyl-(?6)-?-D-glucopyranoside]	7.785
268	6.175	271.16681	Generic	$C_{13}H_{24}N_2O_4$	Elaiomycin D	6.9345
87	4.633	107.05003	Pseudo	C_7H_8O	o-cresol	6.9966
283	4.932	292.08313	Generic	$C_{14}H_{15}NO_6$	Alkaloid AM 6201;Reductiomycin	6.5359

Table S4. Comparative metabolomics profiling of *BPMP-PU-28 (Pseudomonas urmiensis)* and *BPMP-EL-4*0 (*Enterobacter ludwigii*) buffered minimal culture media collected during the stationary phase: top 50 more discriminating metabolites between the two bacterial strains.

For the complete list of metabolites, see Table S2. Corresponding heat map: see Figure 6B. As described in the legends to Figure S5 and Figure 6B, the bacteria were grown in buffered (B) minimal medium, and aliquots of bacterial culture supernatant were collected during the stationary (S) phase (see Figure S5). Numbers at the left refer to the metabolites whose distribution and abundance are reported in the heat map shown in Figure 5B. Annotations were performed using MSCleanR workflow. N°: metabolite number; Rt min: retention time in min; m/z[M+H] +: mass/charge; bank: bank used to identify the metabolites; total score: score of the fragmented metabolite (only metabolites identified with scores equal to or higher than 6.5 were selected for *BPMP-PU-28 (Pseudomonas urmiensis)* (**A**) and *BPMP-EL-4*0 (*Enterobacter ludwigii*) (**B**). Data from three biological replicates, each one being used for two technical replicates.

(A)

N°	Rt.min	m/z [M+H] +	Bank	Formula	Compound annotated	Total score
502	5.374	227.1393	Generic	$C_{11}H_{18}N_2O_3$	Cyclo-(L-Pro-4-OH-L-Leu)	7.0225
51	11.688	310.14432	Generic	$C_{19}H_{19}NO_3$	CJ 16171	7.077
142	9.482	534.2851	Generic	$C_{32}H_{39}NO_6$	SMTP 4D	6.587
289	11.524	312.16	Generic	$C_{19}H_{21}NO_3$	CJ 16170	6.8153
316	6.881	348.10229	Generic	$C_{16}H_{17}N_3O_4S$	Cephalexin	6.7342
476	5.584	761.42029	Generic	$C_{41}H_{56}N_6O_8$	Xentrivalpeptide Q	6.6381
451	8.338	506.25381	Generic	$C_{27}H_{39}NO_6S$	9-Oxoepothilone D	6.794
17	5.511	215.1394	Generic	$C_{10}H_{18}N_2O_3$	Dethiobiotin	7.3222
339	1.624	308.09189	Generic	$C_{10}H_{17}N_3O_6S$	Glutathione	7.4493
10	6.124	275.10382	Pseudo	$C_{14}H_{16}N_2O_4$	Tryptophan; N?-Methoxycarbonyl, Me ester	6.935
246	5.88	280.06161	Generic	$C_{14}H_{13}NO_4$	Exopisiod B	6.6339
27	6.232	216.12376	Generic	$C_{10}H_{19}NO_4$	(2S)-5-acetamidopentyl 2-hydroxypropanoate	7.2705

(B)

N°	Rt.min	m/z [M+H] +	Bank	Formula	Compound annotated	Total score
66	6.534	435.11423	Generic	$C_{24}H_{20}O_8$	Terfestatin C	6.8471
447	9.718	487.14368	Generic	$C_{28}H_{22}O_8$	Streptoanthraquinone A	6.7253
528	5.263	269.06424	Generic	$C_{11}H_{10}N_4O_3$	Columbianetin	7.3162
215	6.794	203.12857	Pseudo	$C_{10}H_{20}O_4$	(3R,9R)-3,9-dihydroxydecanoic acid	6.9589
1	7.555	275.11386	Generic	$C_{12}H_{20}O_7$	Methyl (3r)-3-{[(3r)-3-{[(3r)-3-hydroxybutanoyl]oxy}butanoyl]oxy}butanoate	7.058
288	5.4	305.08792	Pseudo	$C_{12}H_{18}O_9$	Galactose; 1,2,6-Tri-Ac	6.6442
375	6.546	353.15024	Generic	$C_{20}H_{20}N_2O_4$	Hinduchelin D	6.6095
549	10.456	295.15237	Generic	$C_{16}H_{22}O_5$	(1R,2R,3S,8aR)-7-Acetyl-1,2,3,4,8,8a-hexahydro-3,6-dihydroxy-1,8a-dimethylnaphthalen-2-yl acetate	7.0529
505	5.302	118.08648	Generic	$C_{11}H_{19}NO_8$	N-Acetylmuramate	6.5535
535	12.035	277.18051	Generic	$C_{17}H_{24}O_3$	Acremeremophilane H	7.634
497	9.597	214.25349	Generic	$C_{14}H_{31}N$	Medelamine A;NK 148198A	7.3576
519	5.952	273.16797	Pseudo	$C_{14}H_{24}O_5$	Mupirocin H	6.8823
201	9.614	185.11804	Generic	$C_{10}H_{18}O_3$	8-methyl-5-oxo-nonanoic acid	7.2146
223	7.286	217.10777	Pseudo	$C_{10}H_{18}O_5$	3-hydroxysebacate	7.5995
262	10.193	259.19156	Pseudo	$C_{14}H_{28}O_4$	(3R)-3,14-dihydroxytetradecanoic acid	6.9651
351	7.624	315.17844	Generic	$C_{16}H_{26}O_6$	Isoaigialone A	6.5883
219	10.574	211.13373	Generic	$C_{12}H_{20}O_3$	Herbarumin III;(+)-Herbarumin III	6.9954
190	5.581	173.08153	Generic	$C_8H_{14}O_4$	6-Hydroxy-4-oxohexyl acetate	7.1942
403	7.653	387.1636	Generic	$C_{18}H_{26}O_9$	Macrosphelide K	7.1868
514	7.244	245.13892	Generic	$C_{12}H_{20}O_5$	Phomolide G	7.2239
498	6.141	217.10753	Generic	$C_{10}H_{16}O_5$	Aspyronol	7.0509
414	10.748	415.21228	Generic	$C_{24}H_{30}O_6$	Chaetopenoid A	7.1278
450	9.711	501.1954	Generic	$C_{23}H_{32}O_{12}$	Talapolyester D	7.2782
445	9.075	487.17969	Generic	$C_{22}H_{30}O_{12}$	Talapolyester A	7.0784
378	6.782	357.1163	Generic	$C_{16}H_{20}O_9$	Pestalotiopyrone K	7.4888

43	11.924	243.19626	Pseudo	$C_{14}H_{28}O_3$	(+/-)-3-hydroxymyristic acid	6.9071
7	7.053	159.10223	Pseudo	$C_8H_{16}O_3$	(+/-)-3-hydroxyoctanoic acid	8.3247
29	9.028	187.13348	Pseudo	$C_{10}H_{20}O_3$	? -hydroxydecanoic acid	6.842
67	6.062	245.0929	Pseudo	$C_{13}H_{14}N_2O_3$	N-acetyl-D-tryptophan	6.7931
268	6.175	271.1668	Generic	$C_{13}H_{24}N_2O_4$	Elaiomycin D	6.9345
87	4.633	107.05	Pseudo	C_7H_8O	o-cresol	6.9966
348	7.213	313.1266	Generic	$C_{15}H_{20}O_7$	Cyclocalopin A-15-OL	6.746
427	6.836	437.1427	Generic	$C_{21}H_{24}O_{10}$	Daldinin D	6.9164
346	8.306	313.1263	Generic	$C_{15}H_{20}O_7$	Cyclocalopin A-15-OL	7.7123
283	4.932	292.0831	Generic	$C_{14}H_{15}NO_6$	Alkaloid AM 6201;Reductiomycin	6.5359
341	9.204	310.2383	Generic	$C_{18}H_{31}NO_3$	N-Tetradecenoyl-L-homoserine lactone	6.5151
217	10.18	211.1696	Pseudo	$C_{13}H_{22}O_2$	2,4-Tridecadienoic acid	6.5514
317	7.017	371.0959	Pseudo	$C_{14}H_{20}O_{10}$	Mannose; 1,2,3,4-Tetra-Ac	7.2938

Table S5. (Data sheets) List of peptides identified in *BPMP-PU-28 (Pseudomonas urmiensis)* and *BPMP-EL-4*0 (*Enterobacter ludwigii*) or their culture supernatant.

Pseudomonas urmiensis and *Enterobacter ludwigii* were grown in minimal Hoagland medium supplemented with lactate (2%) and buffered with phosphate buffer (150 mM, pH 6.7). Aliquots of culture supernatant or bacteria pellet were collected during the stationary phase.

Data sheet 1: Peptides without digestion
Data sheet 2: Whole bacterial proteome
Data sheet 3: Common culture supernatant and the whole proteome
Data sheet 4: Specifically, protein present in the culture supernatant
